# POLICY SERIES: AGING AND HEALTH POLICY UPDATE: A VIEW FROM WASHINGTON

**DOI:** 10.1093/geroni/igae098.0946

**Published:** 2024-12-31

**Authors:** Brian Lindberg, Patricia D’Antonio

**Affiliations:** Chair; Discussant

## Abstract

Leading policy advocates will present their findings and viewpoints regarding aging and health care policy during a divided Congress. Issues will include behavioral health, social isolation, health care workforce, telehealth, hospice policy, elder justice and nursing home care, home and community-based services, and Medicare and Medicaid.

